# Spatial and Functional Immune Profiling Identifies Impaired Vascular Repair in Human Myocardial Infarction

**DOI:** 10.3390/biomedicines14040755

**Published:** 2026-03-26

**Authors:** Amankeldi A. Salybekov, Saida Shaikalamova, Aiman Kinzhebay, Markus Wolfien, Takayuki Asahara

**Affiliations:** 1 Regenerative Medicine Division, Cell and Gene Therapy Department, Qazaq Institute of Innovative Medicine, Astana 010000, Kazakhstan; a.kinzhebay@qaziim.kz; 2 Department of Advanced Medicine Science, Tokai University School of Medicine, Isehara 2591193, Japan; t.asahara@shonankamakura.or.jp; 3 Shonan Research Institute of Innovative Medicine, Shonan Kamakura General Hospital, Kamakura 2478533, Japan; 4 System Biology and Bioinformatics Department, Qazaq Institute of Innovative Medicine, Astana 010000, Kazakhstan; 5 Institute for Medical Informatics and Biometry, Faculty of Medicine Carl Gustav Carus, TUD Dresden University of Technology, 01307 Dresden, Germany; markus.wolfien@ukdd.de; 6 Center for Scalable Data Analytics and Artificial Intelligence (ScaDS.AI), Strehlener Str. 12–14, 01069 Dresden, Germany

**Keywords:** myocardial infarction, spatial transcriptomics, CD34 cells, immune cells, CD8 cells, endothelial progenitor cells

## Abstract

**Background:** In an earlier murine model of myocardial infarction (MI), we showed that CD8 cells and myeloid dendritic cells (mDCs) infiltrate the infarcted myocardium within the first week. However, in humans, the spatial interplay between CD8^+^ T cells and dendritic cells in the spatial context of human myocardial infarction remains underexplored. **Objective:** In the present study, we applied spatial transcriptomics and functional assays to characterize immune–stromal dynamics in infarcted myocardium and peripheral blood. **Methods & Results:** Spatial transcriptomics analysis of infarcted human myocardium at days 2 and 6 post-MI, combined with peripheral blood flow cytometry and EPC colony-forming assays, was performed. Cell composition, pathway enrichment, and cell-to-cell communication analyses were conducted to map immune–stromal cells’ dynamics across time points. Spatial mapping identified dynamic shifts in immune, fibroblast, and endothelial populations, with fibroblasts and endothelial cells remaining abundant throughout. CD8^+^ T cells accumulated in ischemic regions while their circulating levels declined. Gene Ontology and pathway analyses of CD8A^+^ transcripts revealed enrichment of proinflammatory and NF-κB survival programs. ITGAX/CD33/THBD^+^ APCs progressively increased within infarct zones, activating antigen-presentation and leukocyte chemotaxis pathways. Early (day 2) APC–endothelial crosstalk showed the strongest predicted recruitment signals for CD8^+^ T cells, which diminished by day 6. Finally, EPC colony-forming capacity showed a tendency for reduction in MI patients and inversely correlated with coronary lesion burden, indicating impaired vascular repair potential. **Conclusions:** This integrative spatial and functional study demonstrates that APC-driven CD8^+^ recruitment and EPC dysfunction are key features of human MI. Immune–endothelial niches facilitate early cytotoxic T-cell infiltration, while progenitor depletion limits vascular regeneration. These findings provide mechanistic insight into immune–vascular imbalance during infarct healing and highlight potential therapeutic targets to modulate inflammation and restore vascular repair.

## 1. Introduction

Myocardial infarction (MI) initiates a highly dynamic and spatially orchestrated immune response that critically shapes tissue injury, repair, and remodeling. In the immediate aftermath of ischemia, infiltrating immune cells interact with stromal and vascular populations within the infarcted myocardium, tipping the balance between inflammation and resolution [1]. Recent advances in spatial transcriptomics have provided unprecedented insights into these multicellular interactions. For example, Kuppe et al. integrated single-cell and spatial multi-omics data to generate a comprehensive atlas of the human infarcted heart, revealing distinct myeloid and stromal states across infarct, border, and remote zones and thereby uncovering region-specific mechanisms of injury and repair [2]. Building on this, Ninh et al. identified spatially organized interferon-response clusters (“IFNIC” colonies) in cardiomyocytes at the infarct border, directly linking innate immune activation to maladaptive remodeling [3]. Most recently, Tombor et al. demonstrated that endothelial cells adopt specialized immunoregulatory states and engage in direct crosstalk with immune subsets, highlighting vascular–immune interactions as key drivers of post-MI outcomes [4]. Together, these studies illustrate the power of spatial omics to define cellular niches and communication networks that govern inflammation and repair after MI. Importantly, such approaches not only deepen our understanding of disease mechanisms but also open avenues for identifying novel therapeutic targets aimed at modulating the immune–stromal–vascular axis in cardiac injury.

The adaptive immune response plays a central role in cardiac wound healing following MI, despite the relatively low numbers of T cells in the myocardium [5]. The contribution of T cells to post-MI remodeling is well documented [5,6]. For example, T cells and their subsets have been identified as key effectors of ischemic injury, with experimental ablation significantly reducing myocardial damage in animal models [7]. Conversely, depletion of CD8^+^ T cells impair the clearance of necrotic tissue, leading to defective scar formation and a higher incidence of cardiac rupture, thereby highlighting the dual and context-dependent role of cytotoxic T cells in infarct healing [8]. In other murine studies, myeloid dendritic cells (mDCs) increase in the infarct during the first week, while CD8^+^ T cells progressively accumulate from day 1 through day 28, suggesting that antigen-presenting cells (APCs) orchestrate cytotoxic T-cell recruitment into the injured myocardium [9].

Together, these findings provide a mechanistic rationale to explore whether similar immune–vascular interactions occur in the human setting. However, the integrated spatial and functional interplay of CD8^+^ T cells, APCs, and stromal populations within infarcted human myocardium remains incompletely defined. To address this gap, the present study combines spatial transcriptomics of human infarct tissue with peripheral immunophenotyping and EPC functional assays.

## 2. Materials and Methods

### 2.1. Human Cardiac Tissue Collection and Spatial Transcriptomic Profiling

This study utilized spatial transcriptomic data from infarcted and donor human heart tissues, originally generated by Kuppe et al. [2], in which human cardiac tissue samples were obtained from the left ventricle, specifically from the ischemic infarct zone, border zone, and remote (non-infarcted) zone of patients who died or underwent cardiac surgery following MI. The donor (control) heart tissues were also obtained from the left ventricle of organ donors without prior cardiac disease. Briefly, samples were snap-frozen in liquid nitrogen, embedded in OCT compound (Tissue-Tek, Sakura Finetek, Torrance, CA, USA), and cryosectioned using a CryoStar NX70 (Thermo Fisher Scientific, Waltham, MA, USA). Hematoxylin and eosin (H&E) staining was performed to enable pathological assessment and region selection by a cardiac pathologist. RNA quality was evaluated from parallel samples using the RNeasy Mini Kit with the fibrous tissue protocol (Qiagen, Hilden, Germany) and Bioanalyzer RNA 6000 Nano Kit (Agilent Technologies, Santa Clara, CA, USA). Spatial transcriptomic profiling was carried out using the 10× Genomics Visium Spatial Gene Expression platform (10× Genomics, Pleasanton, CA, USA). Tissue permeabilization was optimized using Visium Optimization Slides (10× Genomics, Pleasanton, CA, USA), and library preparation was performed according to the manufacturer’s protocol, which included reverse transcription, second-strand synthesis, and cDNA amplification. Brightfield histology images were acquired using Nikon Eclipse Ti-E (Nikon Co., Ltd., Osaka, Japan) and Leica Aperio Versa 200 systems (Wetzlar, Germany), with image stitching performed in NIS-Elements software (v1). Sequencing libraries were quantified, loaded, and sequenced on an Illumina NovaSeq 6000 system (Illumina Inc., San Diego, CA, USA).

### 2.2. Spatial Data Preprocessing and Analysis

Raw count matrices from Space Ranger (v1.3.2, 10× Genomics, Pleasanton, CA, USA), employing the GRCh38 reference genome, were imported into Python (version 3.13) and analyzed using the Scanpy library (v1.10.3) [10]. Downstream analyses of MI tissue samples [2], including normalization, scaling, and dimensionality reduction, were performed using Seurat (v5.1.0, R). To correct for inter-sample batch effects across different time points and patients, Harmony (v1.2.3) [11] was applied. Gene identifiers were converted from Ensembl IDs to HGNC gene symbols prior to analysis. Data were normalized to counts per spot, log-transformed, and scaled. A k-nearest neighbor graph was constructed, followed by Leiden clustering and Uniform Manifold Approximation and Projection (UMAP) algorithm, applied using the scanpy.tl.umap function.

### 2.3. Cell Annotation

Clustered cell populations were annotated through a combination of automated predictions using reference transcriptome atlases, specifically Azimuth [12] and CellTypist (version 1.6.3) [13], and manually curated reference gene lists for fibroblasts, endothelial subtypes, and cardiomyocyte populations, based on canonical markers from published literature. To characterize the functional roles of specific cell populations, GO biological process and pathway enrichment analysis was performed using Enrichr [14]. Enrichment results (*p* < 0.05) were used to infer the cellular processes and signaling pathways most associated with each cell type.

### 2.4. Ligand–Receptor Analysis

Cell–cell interactions were examined using Cellphone DB (v 5.0) in Python (v3.13) [15]. The CellphoneDB human ligand–receptor database was used to identify potential communication pathways between cell clusters. The frequency and strength of interactions between cell type pairs were quantified across different experimental conditions (days 2 and 6). Cell–cell communication networks were visualized using Cellphone’s built-in visualization functions, including chord diagrams generated with netVisual_aggregate() to display the interaction counts and communication strength between different cell populations.

### 2.5. Subjects Enrollment

To provide functional validation, we included a prospectively enrolled cohort of eighteen patients admitted to Tokai University Hospital (Kanagawa, Japan) with a diagnosis of acute myocardial infarction (AMI). Patients with chronic inflammatory disease or those receiving anti-inflammatory therapy were excluded. Myocardial infarction was defined by a rise and fall of cardiac troponin T (cTnT) and at least one of the following: ischemic symptoms, new ST-T changes, or pathological Q waves. Immediately after hospitalization, patients underwent emergency coronary angiography and percutaneous coronary intervention with drug-eluting stent placement. Optical coherence tomography was performed when indicated to guide stent deployment and evaluate coronary pathology. All patients received standard AMI pharmacotherapy (aspirin, P2Y12 inhibitors, heparin, beta-blockers, ACE inhibitors/ARBs unless contraindicated). Sixteen healthy volunteers under 60 years of age, without cardiovascular disease or diabetes, and without a family history of dyslipidemia or AMI, were enrolled as controls.

### 2.6. Specimen Collection and Cell Phenotype Characterization

Peripheral blood (30–40 mL) was collected by venipuncture at days 2 and 6 after AMI onset and from healthy controls. Peripheral blood mononuclear cells (PBMCs) were isolated by density gradient centrifugation (Histopaque, Sigma-Aldrich, St. Louis, MO, USA). After separation, red blood cells were lysed, platelets were removed by two additional centrifugation steps, and viable PBMCs counted. Flow cytometry was used to characterize circulating cell populations. Cells were incubated with Fc receptor block and stained with antibodies against CD34, CD133, CD11b, and CD11c (BioLegend, San Diego, CA, USA). Propidium iodide was used as a viability marker. Stained cells were acquired using BD FACSVerse or LSR Fortessa instruments (BD Biosciences, San Jose, CA, USA) and analyzed with FlowJo (v10.6).

### 2.7. EPC Colony-Forming Assay

Freshly isolated PBMCs (1.5 × 10^5^) were seeded into methylcellulose-based culture dishes and incubated at 37 °C, 5% CO_2_ until colonies appeared. Primitive (PEPC-CFU) and definitive (DEPC-CFU) EPC colonies were counted on days 16–18 under a phase-contrast microscope, following previously established protocols [16,17].

### 2.8. Statistical Data Analysis

All values are shown as mean ± SE. Statistical differences were investigated using One-way ANOVA and followed by Tukey’s post hoc analysis for multiple-time-point comparison. Correlations were evaluated using the Spearman rank correlation test. All statistical analyses were performed using the GraphPad Prism Software (v.10 GraphPad, San Diego, CA, USA). Statistical significance was given by *p* < 0.05. 

## 3. Results

### 3.1. Spatial Annotation of the Infarcted Myocardium Reveals Dynamic Shifts in Cell Composition

**Figure 1:**
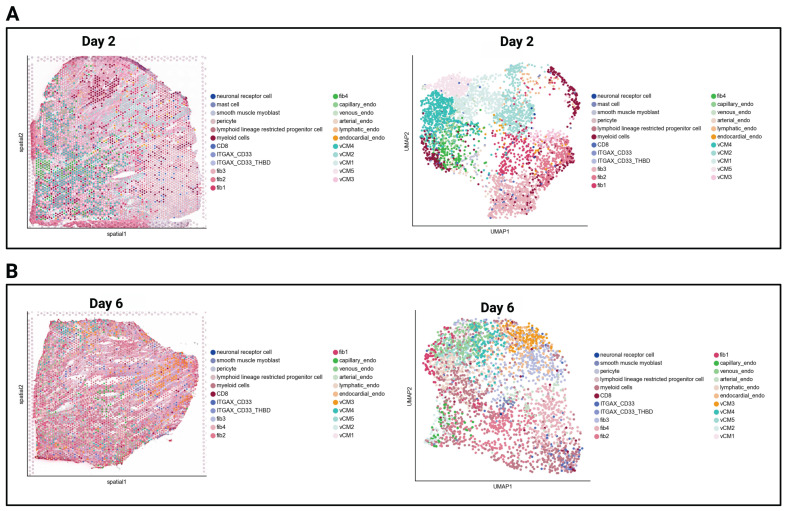
Spatial annotation of the infarcted myocardium reveals dynamic shifts in cell composition. Spatial transcriptomic maps (**left panels**) and corresponding UMAP plots (**right panels**) showing annotated cell populations in infarcted human myocardium at Day 2 post-MI (**A**) and Day 6 post-MI (**B**). Each spot on the tissue section represents a 10× Genomics Visium capture spot (55 µm diameter), colored by assigned cell type. Cell type annotations were performed using a combination of Azimuth, CellTypist, and manually curated marker gene sets. Major populations identified include CD8^+^ T cells, ITGAX/CD33/THBD^+^ myeloid antigen-presenting cells, fibroblast subsets (fib1–fib4), ventricular cardiomyocytes (vCM1–vCM4), capillary/venous/arterial endothelial cells, lymphoid lineage progenitor cells, smooth muscle myoblasts, and neuronal receptor cells.

Spatial transcriptomic profiling of infarcted human myocardium using the 10× Genomics Visium platform (Figure 1A,B enabled a detailed annotation of immune, stromal, and vascular populations across days 2 and 6 post-MI). The dataset captured diverse cell types, including lymphoid lineage progenitors, CD8^+^ T cells, ITGAX/CD33/THBD^+^ myeloid cells, fibroblast subsets, cardiomyocytes, and endothelial cells (capillary, venous, and arterial). Quantitative analysis revealed marked shifts in the relative abundance of these populations over time. Immune cell clusters, including CD8^+^ T cells and dendritic-like myeloid cells, were enriched within ischemic and peri-ischemic zones. Fibroblasts and endothelial cells remained the most abundant cell classes at all time points, reflecting their central roles in matrix deposition and vascular remodeling during infarct healing.

### 3.2. CD8^+^ T Cells Accumulate in Infarct Tissue, Accompanied by Decreased Levels in Peripheral Blood

**Figure 2:**
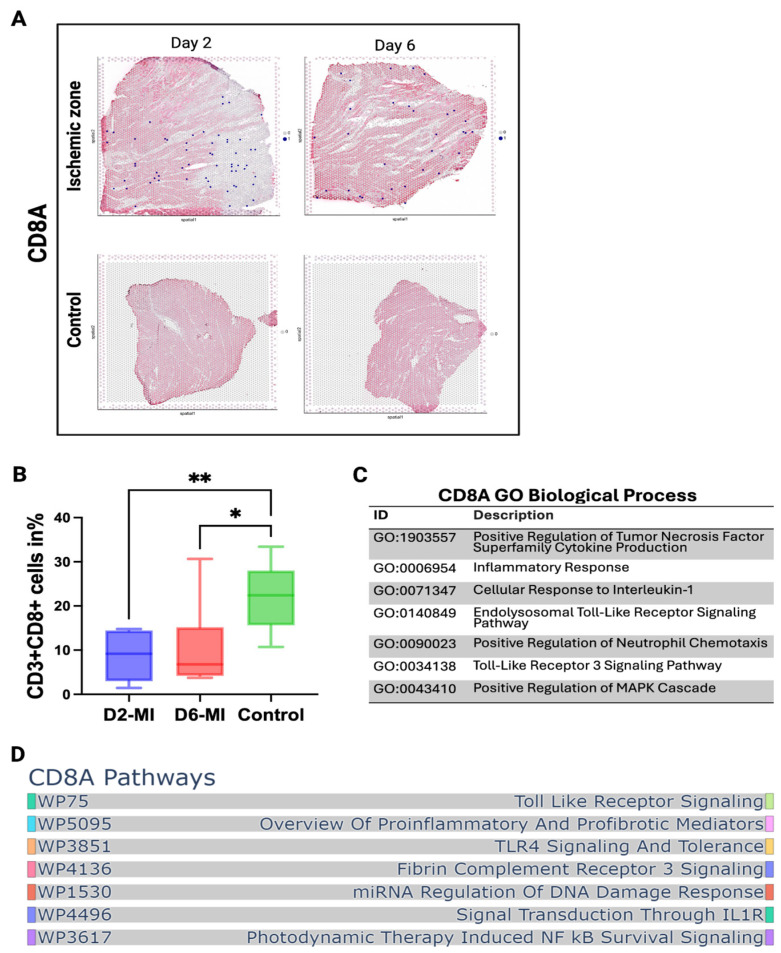
Spatial and functional profiling of CD8^+^ T cells in infarcted cardiac tissue. (**A**) Spatial transcriptomics map showing CD8^+^ cell spots across infarcted cardiac tissue. Each spot represents a Visium spot, capturing transcripts from a group of neighboring cells. Control samples were patients who underwent for cardiac transplantation. (**B**) Quantification of CD8^+^ spatial spots at days 2 and 6 post-MI. (**C**) Percentage of CD3^+^ CD8^+^ cells within total PBMCs across days 2 and 6 in peripheral blood. (**D**) CD8A GO biological processes pathway enrichment analysis. In the graph, * and ** depicts *p* < 0.05 and *p* < 0.01, respectively.

Spatial transcriptomic analysis revealed clear enrichment of CD8A^+^ cells spatial spots, showing that infiltration was the highest at day 2, and remained elevated at day 6 within ischemic myocardial tissue compared with control samples, which did not have any expression at all (Figure 2A). In contrast, peripheral blood flow cytometry demonstrated a significant decrease in circulating CD8^+^ T cells following MI (Figure 2B). To further characterize the functional profile of infiltrating CD8^+^ cells, enrichment analyses were performed. Gene Ontology biological process analysis of CD8A-associated transcripts highlighted proinflammatory programs including regulation of TNF superfamily cytokine production, cellular responses to interleukin-1, and positive regulation of neutrophil chemotaxis (Figure 2C). Pathway enrichment analysis revealed upregulation of signaling cascades related to Toll-like receptor signaling, DNA damage responses, and NF-κB survival pathways (Figure 2D). Together, these results suggest that reduced peripheral CD8^+^ T cell counts reflect recruitment into the infarcted myocardium, where they engage in inflammatory and stress-response programs that may contribute to tissue injury.

### 3.3. Antigen-Presenting Cells Recruit CD8^+^ T Cells via ITGAX-Associated Signaling

**Figure 3:**
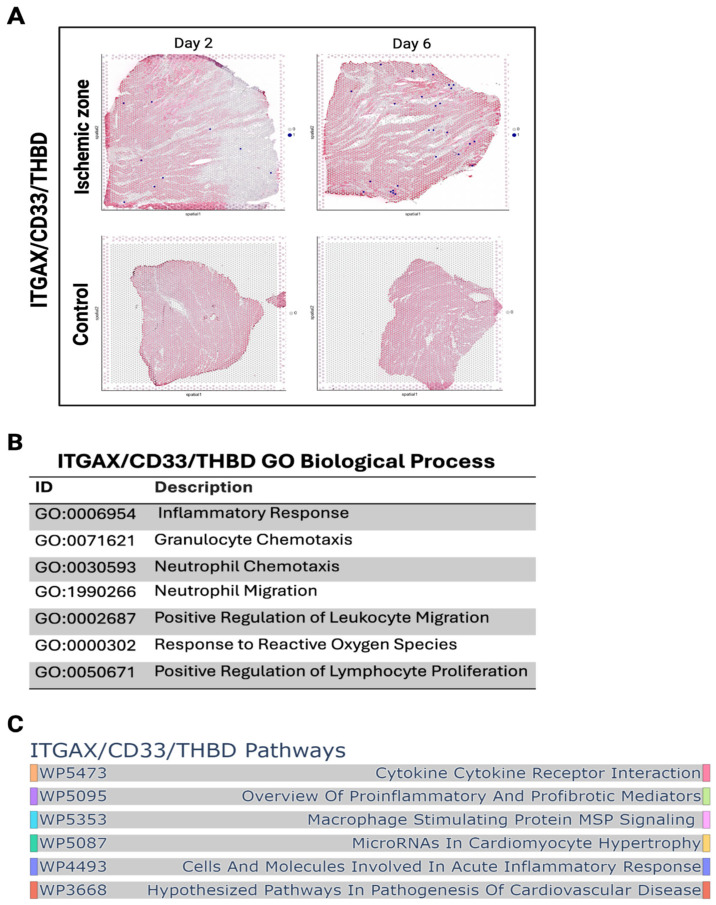
(**A**) Spatial transcriptomics map showing ITGAX/CD33/THBD cell spots across infarcted cardiac tissue. Each spot represents a Visium spot, capturing transcripts from a group of neighboring cells. (**B**) Quantification of ITGAX/CD33/THBD spatial spots at days 2 and 6 post-MI. (**C**) ITGAX/CD33/THBD GO biological processes pathway enrichment analysis.

To explore the mechanisms underlying CD8^+^ T cell infiltration, we examined APC signatures in the same dataset. Spatial mapping specifically assessed myeloid cells defined by the ITGAX (CD11c), CD33, and THBD biomarker set, revealing their enrichment in infarct zones compared with controls (Figure 3A). Quantification of spatial spots confirmed progressive accumulation of these APCs, with the highest numbers observed at day 6 post-MI (Figure 3B). Enrichment maps indicated activation of antigen presentation and immune synapse pathways (Figure 3C). GO biological process enrichment of ITGAX/CD33/THBD-associated transcripts revealed activation of inflammatory pathways, including granulocyte chemotaxis, neutrophil migration, leukocyte trafficking, and responses to reactive oxygen species (Figure 3C). Complementary pathway analysis highlighted cytokine–cytokine receptor interaction, proinflammatory mediator signaling, and acute inflammatory response pathways (Figure 3C). These findings indicate that myeloid APCs become progressively enriched in ischemic myocardium after MI and upregulate proinflammatory signaling cascades. The presence of activated ITGAX^+^ dendritic cells and CD33^+^/THBD^+^ myeloid cells likely contribute to the recruitment and retention of CD8^+^ T cells in infarcted regions, providing a mechanistic explanation for their unexpected accumulation in human cardiac tissue.

### 3.4. APC–Endothelial Crosstalk Suggests Impaired CD8^+^ Cell Recruitment

**Figure 4:**
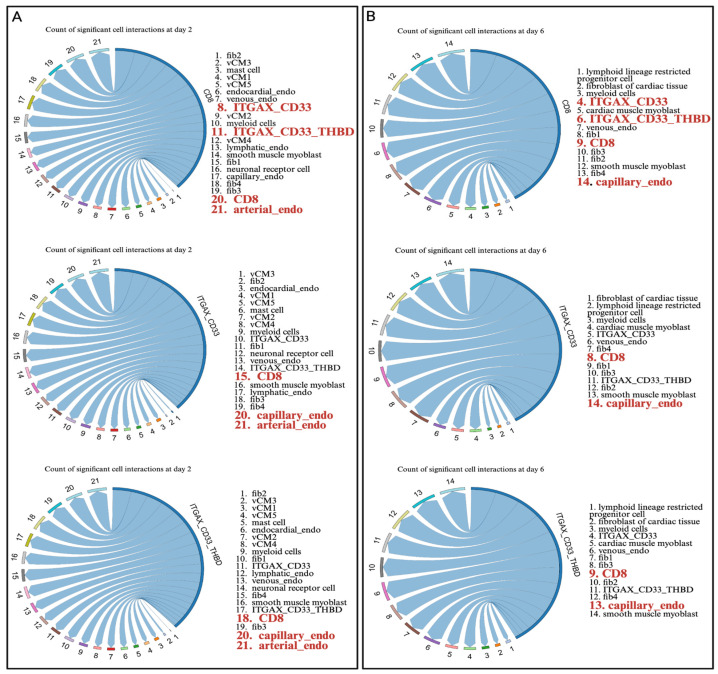
Cell–cell communication analysis reveals dynamic immune–endothelial interactions after MI at days 2 (**A**) and 6 (**B**). Ligand–receptor connections are ordered from weakest to strongest, with arrow size proportional to interaction strength.

At day 2 post-MI, CD8^+^ T cells showed the highest predicted communication strength with endothelial cells and with each other, suggesting rapid intravascular activation and local clustering at the injury site. CD8^+^ cells also displayed moderate interactions with fibroblasts, consistent with their localization near necrotic or remodeling zones. Notably, ITGAX/CD33/THBD^+^ myeloid dendritic cells exhibited their strongest ligand–receptor signaling with endothelial subsets at day 2, including both capillary and arterial endothelium (highlighted in red in Figure 4). These myeloid cells also ranked among the top predicted interactors with CD8^+^ T cells at this early time point, suggesting that APC–CD8 crosstalk is most pronounced during the initial inflammatory phase. By day 6 post-MI, the predicted APC–CD8 interaction strength decreased, even though endothelial interactions persisted, indicating that active CD8 recruitment is likely concentrated in the first few days after infarction. Together, these data support a model in which endothelial-APC signaling creates a transient chemotactic niche that facilitates early CD8^+^ T cell infiltration, with diminishing recruitment as the infarct progresses toward resolution.

### 3.5. The Tendency of EPC Colony Formation Is Reduced and Negatively Correlates with Lesion Burden

**Figure 5:**
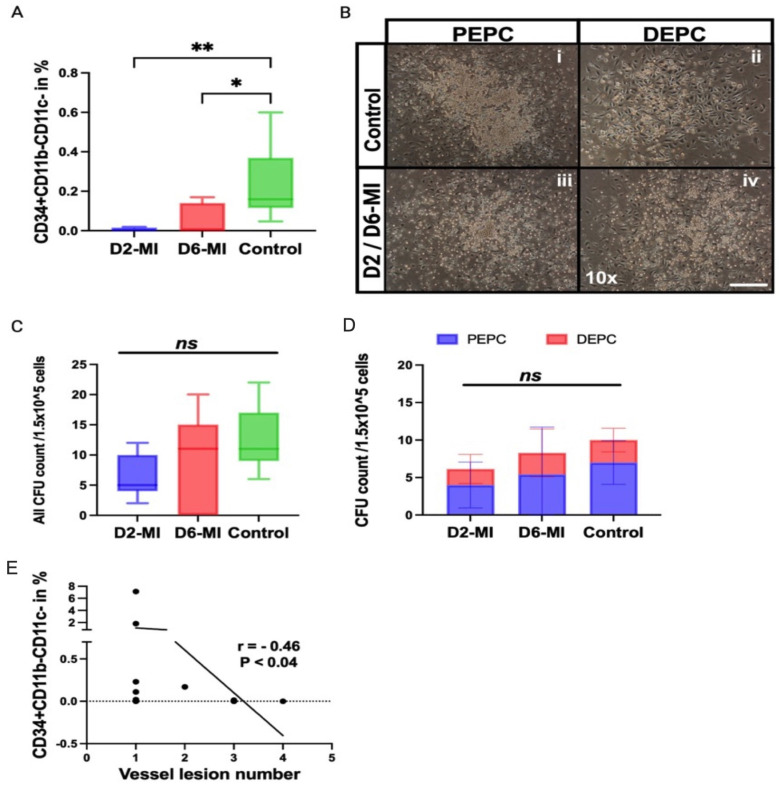
Endothelial progenitor cell frequency is reduced after myocardial infarction and inversely correlates with coronary lesion burden. (**A**) Box plot showing the frequency of circulating endothelial progenitor cells (EPCs). (**B**) Representative phase-contrast microscopy images (10× magnification; scale bar shown) of EPC colony morphology under two culture conditions: proliferative EPC colonies (PEPC, **left column**) and definitive EPC colonies (DEPC, **right column**). (**C**) Total colony-forming unit count per 1.5 × 10^5^ cells across D2-MI, D6-MI, and Control groups. (**D**) Stacked bar graph depicting CFU counts stratified by colony subtype—PEPC (blue) and DEPC (red)—per 1.5 × 10^5^ cells across the three groups. (**E**) Scatter plot illustrating the correlation between CD34^+^CD11b^−^CD11c^−^ EPC frequency (%) and vessel lesion number in MI patients. In the graph, * and ** depicts *p* < 0.05 and *p* < 0.01, respectively.

The total number of circulating immature EPCs (CD34^+^ CD11b^−^ CD11c^−^) at day 2 was dramatically reduced compared with control groups (Figure 5A). Notably, in some patients, immature EPCs were completely absent. By day 6, however, an increased number of immature EPCs were observed in certain patients, which may reflect a mobilization response following ischemic injury. To further evaluate the functional capacity of EPCs, we performed an endothelial progenitor cell colony-forming assay (EPC-CFA) to quantify primitive (PEPC) and definitive (DEPC) colony-forming units (CFUs) (Figure 5B). Figure 5B is a representative example from MI patients assessed at either day 2 or day 6, as colony morphology was qualitatively similar between these two time points. Control cultures exhibited slightly higher colony density, with PEPC and DEPC colonies maintaining the expected morphology (Figure 5B). In particular, DEPC colonies in controls showed the characteristic cobblestone-like appearance indicative of a mature endothelial phenotype capable of initiating vascularization (Figure 5B). By contrast, post-MI cultures were less dense, with PEPC and DEPC colonies displaying a more uniform distribution and predominantly small, rounded cells, consistent with reduced maturation and a shift toward a more proliferative, less differentiated state (Figure 5B). PEPC and DEPC EPC-CFU numbers showed a considerable decrease on day 2 compared to the control, with partial recovery on day 6. However, these changes did not reach statistical significance when analyzed either collectively (Figure 5C) or separately (Figure 5D). To further examine the relationship between EPC biology and vascular pathology, circulating CD34^+^ CD11b^−^ CD11c^−^ EPC frequencies were compared with coronary lesion burden (Figure 5E). Correlation analysis revealed a significant inverse association (r = −0.46, *p* < 0.04), suggesting that EPC dysfunction in lesion-prone individuals is not confined to a specific colony subtype but instead reflects a broader impairment of vascular progenitor activity. Collectively, these findings indicate that EPC depletion and functional decline are correlated, potentially contributing to inadequate vascular repair capacity during the early phase after myocardial infarction.

## 4. Discussion

This study delineates an early, spatially confined immune–stromal cell program in human MI, in which APC enrichment and APC-endothelial crosstalk precede and likely enable CD8^+^ T-cell infiltration, while a concomitant deficit in EPC function constrains vascular repair. In infarcted human myocardium at days 2 and 6, we observed temporally dynamic niches of ITGAX/CD33/THBD^+^ myeloid cells and CD8^+^ T cells concentrated in ischemic and peri-ischemic regions, whereas fibroblasts and endothelium remained abundant across time, consistent with matrix remodeling and angiogenic demands during healing.

Mechanistically, our data align with and extend mouse-to-human insights. In mice, mDCs rise within the first week and temporally coincide with progressive CD8^+^ accumulation through day 28, implicating APCs as gatekeepers of cytotoxic recruitment [9]. We observe a similar axis in human tissue: spatial enrichment of ITGAX^+^/CD33^+^/THBD^+^ APCs, activation of antigen presentation and chemotaxis programs, and early (day 2) ligand–receptor coupling to endothelial and CD8^+^ compartments, followed by waning APC–CD8 signaling by day 6. These findings are conceptually similar to those in murine data with human spatial readouts and are consonant with reports that dendritic cells prime autoreactive T cells after sterile myocardial necrosis [9]. The CD8^+^ T-cell phenotype captured enrichment of TNF-superfamily regulation, IL-1 responses, and TLR/NF-κB survival pathways, which suggests that CD8^+^ cells play a context-dependent role in infarct healing. Ablation studies demonstrate that removing CD8^+^ T cells can blunt early injury yet impair clearance of necrotic tissue and scar maturation, increasing rupture risk; our tissue infiltration with peripheral depletion supports active trafficking from blood to myocardium during this window [8]. In earlier studies, increased numbers of total CD3^+^ T cells and CD8^+^ cytotoxic T cells were detected in human heart failure (HF) patients’ myocardial tissue, with more CD3^+^ and CD8^+^ cells in 5 out of 7 (71%) ischemic HF patients compared with donors, confirming the relevance of myocardial CD8^+^ T cells not only in AMI phase also chronic phase or HF [18]. These results also indicate that increased myocardial T cell numbers are not a simple bystander effect of total immune cell infiltration into damaged tissue, but active infiltration into the otherwise healthy myocardium.

We observed marked quantitative and qualitative dysfunction of EPCs in the peripheral blood of MI patients. Circulating immature CD34^+^ CD11b^−^ CD11c^−^ EPCs were significantly reduced at day 2 and day 6 compared with healthy controls, and although partial recovery was noted by day 6, levels remained markedly below baseline. Functional assays further confirmed that both primitive and definitive EPC colony-forming units were impaired, indicating a limited regenerative potential of mobilized EPCs. Primitive EPCs (PEPCs), which form small-sized colonies, displayed higher proliferative activity and a greater proportion of cells in S-phase compared with definitive EPCs (DEPCs) that form larger colonies; however, DEPCs exhibited significantly greater vasculogenic capacity, with enhanced adhesive properties and the ability to form tube-like structures in vitro. These findings are consistent with a previous study in diet-induced obese mice with hind-limb ischemia, which reported a higher proportion of primitive EPCs than definitive EPCs in peripheral blood and showed that DEPC functional quality was impaired [16]. Similarly, in our MI patients, EPC colony density and DEPC functionality were markedly reduced. Importantly, the frequency of circulating EPCs was inversely correlated with coronary lesion number (r = −0.46, *p* < 0.04; Figure 2D), suggesting that EPC depletion is particularly pronounced in individuals with higher atherosclerotic burden. Collectively, these observations extend previous reports linking EPC quantity and biological quality to vascular outcomes, including associations with atherosclerotic progression and adverse cardiovascular events, and reinforce the concept that limited vasculogenic reserve may exacerbate post-MI remodeling [19].

The main limitations of the study are related to the patient sample size, and future validation in larger cohorts will be valuable to strengthen the generalizability of our findings. In addition, the spatial transcriptomic and functional EPC analyses were performed in different patient groups, acknowledging that infarct size heterogeneity may influence immune cell counts and did not allow for direct correlation between molecular signatures and cellular function. The future studies with patient-matched correlation between systematic infarct size quantification (e.g., CMR), spatial transcriptomics and functional EPC analyses will be needed to fully address this confounding factor.

## 5. Conclusions

The conclusions drawn are based on biological plausibility and internal consistency with prior murine literature rather than on direct patient-matched correlation between tissue transcriptomics and circulating EPC function. Nonetheless, the complementary nature of these approaches provides coherent insights, and the observed patterns are consistent with prior murine studies where APC–CD8 interactions were shown to play an important role in post-MI remodeling [9]. Our results extend these mechanistic insights to the human setting, supporting their translational relevance.

## Data Availability

All research data are available in this manuscript.
